# Long-term memory effects on working memory updating development

**DOI:** 10.1371/journal.pone.0217697

**Published:** 2019-05-31

**Authors:** Caterina Artuso, Paola Palladino

**Affiliations:** 1 University of Urbino, Urbino, Italy; 2 University of Pavia, Pavia, Italy; Educational Testing Service, UNITED STATES

## Abstract

Long-term memory (LTM) associations appear as important to cognition as single memory contents. Previous studies on updating development have focused on cognitive processes and components, whereas our investigation examines how contents, associated with different LTM strength (strong or weak), might be differentially updated at different ages. To this end, we manipulated association strength of information given at encoding, in order to focus on updating pre-existing LTM associations; specifically, associations for letters. In particular, we controlled for letters usage frequency at the sub-lexical level. We used a task where we dissociated inhibition online (i.e., RTs for updating and controlling inhibition from the same set) and offline (i.e., RTs for controlling inhibition from previously updated sets). Mixed-effect analyses were conducted and showed a substantial behavioural cost when strong associations had to be dismantled online (i.e., longer RTs), compared to weak ones; here, in primary school age children. Interestingly, this effect was independent of age; in fact, children from 7–8 to 9–10 years were comparably sensitive to the strength of LTM associations in updating. However, older children were more effective in offline inhibitory control.

## Introduction

Working memory (WM) is a capacity limited system, able to maintain actively sets of representations useful in complex cognitive skills such as reading [1, 2] or text comprehension [3, 4]. WM performance improves substantially over childhood with linear increases [5, 6]. These developmental improvements may be driven by increases in storage capacity [7], rehearsal strategies [8], or also updating processes [9].

In fact, given capacity limits and the continuous flow of information to be processed, it is important to explore a mechanism that potentially allows WM content to be updated constantly via maintenance of relevant information and inhibition of irrelevant information. Updating investigation is usually applied to memory contents [10]. However, usually, updating tasks are based on binding and unbinding processes between memory contents (e.g., [11]). Binding updating (but not content updating) is a more sensitive measure in accounting for performance in accuracy-based updating tasks [12]. In addition, the role of associative contextual bindings in episodic memory retrieval was also supported [13]. Overall, it appears that the monitoring of associative bindings between contents is a specific challenge for the updating process (see also [14, 15]).

In the current paper, we aimed to study how updating of long-term memory (LTM) bindings (or LTM associations) develops in primary school children (in particular from third to fifth grade). First, we briefly review development of updating components and the role of LTM representations in WM tasks through childhood; in particular, lexical-semantic and sub-lexical representations. Next, we will focus on sub-lexical LTM representations and how these are updated specifically, introducing the aims of the current study.

### Updating processes, components and development

Development of the WM updating function is a recent research topic that has arisen from adult studies and modelling research. In a recent developmental study, an accuracy-based updating task modelled after the one developed by [4] was administered to children [9]; here, they were able to differentiate between inhibition (i.e., ability to suppress same-lists intrusions) and proactive interference (PI) control (i.e., ability to suppress previous-lists intrusions). They showed that memory performance improves with age, together with development of inhibitory process efficiency. However, the most interesting finding here, is that these two components are relatively dissociable. The inhibition of information explained a considerable amount of variance, but of a similar percentage magnitude at ages 7, 11 and 15 years (42%, 49% and 46%, respectively); thus, its developmental contribution is less pronounced. On the other hand, the PI control component explained smaller amounts of variance across all ages, but especially at 7 years (25%), at 11 years (17%) and at 15 years (13%; [9]); thus its developmental role appeared more pronounced.

This two-component model of updating development is consistent with other models that emphasize additional features of updating and/or investigate alternative mechanisms [16]; here, the authors decomposed the updating process, individuating at least three components: retrieval (i.e., searching for a specific representation among many competing elements maintained in the region of direct access; see also [17]); transformation (i.e., modifying a representation maintained in WM); and the most distinctive component, item-removal (i.e., replacement of previously relevant content -now irrelevant- with new relevant information; [16, 18]).

Within this theoretical framework, age-related differences through development, from 8 years to adulthood were found [19]. They found that only the retrieval component has age-related effects, with clear development from 8 years; no differences were observed for transformation or item-removal, despite their crucial role in updating.

### LTM associations and WM development

The role of LTM associations in WM performance has been previously explored in order to understand how enduring properties of verbal material affects ongoing performance, mainly through simple WM tasks involving recall (e.g., [20, 21]). The impact of informational organization in LTM on WM performance can be observed at different processing levels, e.g., lexical, sub-lexical and semantic.

In general, it has been shown that LTM associations interact with recall, facilitating the process; the more strongly items are associated in LTM, the more WM performance will benefit. That said, few studies have investigated the influence of lexical/semantic LTM representations on verbal WM performance in children, although previous research seems to suggest that effects are similar in children and adults (e.g., [22, 23, 24]).

Semantically-related information enhanced WM performance more than descriptive or unrelated information [22]. Similar lexico-semantic effects to adults across development were reported [23]. In an immediate serial recall task with words, they found replication of effects observed in adults, (e.g., lexicality, word frequency and imageability) from 6 to 22 years. These were accounted for by similar redintegration processes that would operate effectively on high frequency words because their phonological representations are more easily accessed by partial information. Accordingly, item frequency effects on recall are observed with the relevant item only, and occur at the time the individual item is retrieved/recalled (see also [20, 21, 25]).

How LTM lexical/semantic knowledge (such as lexicality and language familiarity effects) impacts on WM performance was examined by [24]. They compared children aged 5 and 9 years in tasks of immediate serial recall, finding evidence of the semantic-similarity effect in 5 year-olds. In fact, the specific organization of semantic LTM was found to enhance recall performance.

Overall, these studies have focused on WM recall tasks (i.e., entailing temporary maintenance of information in WM; [2]) and suggest that the more associated the information is, the better memory performance will be. In addition, studies suggest that developmental changes of the LTM system happens between the age of 5 and 11 years [24]; thus, interactions between LMT and WM recall are linked to developmental changes in WM capacity and efficiency [6]. In contrast, here, we focused on the interaction between LTM and updating; here, a complex WM function comprising not only temporary maintenance of information, but also inhibition and item-removal [9, 16, 18].

### How LTM associations are updated

To the best of our knowledge, few studies have investigated the updating of LTM associations between verbal materials [14, 26]. Indeed, updating can be distinguished from recall, as it allows memory focus to remain attuned to the most relevant information in any specific moment.

In an initial study, the strength of association between LTM stimuli was manipulated [26]; and how strength might modulate the updating process itself. Following the literature on the beneficial effects of highly-associated LTM information (e.g., [20, 25]), Artuso and Palladino [26] investigated whether strong or weak associations were updated differently. Strength was represented by the frequency of sub-lexical associations between consonants. Association strength was manipulated at encoding, in order to observe how strong and weak associations were updated subsequently. Overall, it was shown that the stronger the LTM association, the longer latencies (i.e., to substitute information and to control for irrelevant information) were required. Therefore, a processing cost was found for updating; this is in direct opposition to recall, which is boosted by association strength [14].

In a further study, the association strength was manipulated at both encoding and updating, and added two conditions (i.e., strong associations that were updated to strong, and weak associations updated to strong), in order to gain a more complete view of accumulation and disruption of specific associations [14]. Here, the data supported the view that as pre-existing associations became stronger, they became harder to dismantle (i.e., longer RTs). In addition, when a strong association had to be recreated, this was usually enhanced (i.e., with shorter RTs from weak to strong association). The result was observed for both processing speed (inhibition process) and interference control (i.e., of a previously activated strong association). In particular, it was shown that inhibitory control requested was greater for items strongly associated, indicating, in turn, the long lasting of the pre-existing LTM association. Those experiments demonstrated clearly that associations from LTM modulate the updating process. In fact, these results suggested that, on the one hand, strong associations are dismantled and updated with greater difficulty (i.e., they require longer RTs), and on the other, that strong associations are activated more easily (i.e., requiring shorter RTs). This evidence supported the idea that the nature of updating rests in the interplay between dismantling and reconstructing bindings via different memory systems such as WM [11, 24] and episodic LTM [13].

In the numerical domain, it was found that numerical similarity produces facilitation during updating of information. When the numbers involved in updating were near as far as concern numerical distance, or similar through sharing a digit, substitution occurred more quickly [27, 28]. There, it was proposed that updating is a function of the overlapping features [29] between numbers to update and those stored in LTM; the greater the amount of overlap, the quicker the update will be, as both numbers share many (already activated) features. In summary, if, as well as inhibition [9], item-removal in LTM association is a distinctive updating component [16], it is important to investigate how the strength of this inter- item association retained in LTM affects WM processing (e.g., updating, [14]).

### The current study

As previously described, studies on updating development have focused on processes and components [9, 19], whereas our aim is to examine the associative effects of updating through development. In particular, given that LTM inter-item associations seem as important as single contents [14], we aimed to investigate whether associated information modulates updating performance in development.

Hence, we manipulated LTM associations for letters as they represent initial elements of learning and therefore, should be highly familiar to children, in addition to their established use in many studies on their role across cognition. In particular, we controlled for their frequency of use at the sub-lexical level. Broad evidence has shown recall accuracy is greater for words containing high frequency phoneme combinations in English (“phonotactic effect”, see [25]). Performance would likely benefit from use of stored phonotactic representations for familiar words to fill in incomplete traces prior to output. In contrast, for unfamiliar words, no stored representations are available to reconstruct partial traces, and this will lead to diminished accuracy at recall. In addition, recall is better for high phonotactic frequency of the language in WM. As fully described in [25] the “phonotactic effect” elicits better recall for ‘consonant-vowel-consonant’ non-words containing ‘consonant-vowel’ and ‘vowel-consonant’ combinations, common in the language’s native phonology, than for non-words containing low probability ‘consonant-vowel’ combinations. Such effect would reflect the influence of phonotactic knowledge about properties of that language [25].

With this aim, we administered an updating task previously used with both children [30] and adults [12, 31], focused on active item-removal of information shown to be the most distinctive component of updating [14, 16]; but see also [19]. In particular, this task allows collection of both online response times (RTs) during updating (i.e., dismantling of an item-set) and offline accuracy/RTs after updating of a memory set, in order to ensure updating effectiveness and inhibition of irrelevant information [31].

Therefore, we believe this task could include at least two different types of inhibition, that is online (i.e., inhibition within the same set) as well as offline (i.e., inhibition of the previously updated set of information). Thus, the specific object of our investigation is how information, associated with different strength in LTM, i.e., strongly or weakly, might be differently updated at various ages. To this end, we manipulated association strength of the information at encoding (but not updating), in order to focus on the specific function of dismantling pre-existing LTM associations rather than reconstruction of new associations. We hypothesize that, in line with adult studies (e.g., [23]), we should observe similar effects with children, as soon as LTM representations are strengthened and consolidated (i.e., with a behavioural cost for updating strongly associated information). In particular, we should observe an increase in online updating RTs when inhibiting and dismantling a strong pre-existing association (once encoded), and a decrease when dismantling a weak pre-existing association (once encoded). Accordingly, offline, we predict greater difficulty in inhibiting items from strong LTM associations, relative to weak ones).

## Methods

### Participants

The initial sample was of 90 children. At the end of the testing phase, we were informed from teachers that one child had received a diagnosis of learning disorder. We therefore decided to not include his data in the final sample. Thus, a sample of 89 children took part in the study. They did not present any specific learning, neurological or psychiatric disorder. Children were divided into two groups: 44 younger children (aged 7–8 years) and 45 older children (aged 9–10 years). These specific ages were chosen as they represent the most crucial steps for children to become more and more skilled in reading and writing, and access to meaning of written texts is more automatized. In addition, and in line with previous studies suggesting the relevance of the specific age range 5–11 years (e.g., [6, 24]), we chose two central and crucial steps that are coherent with previous research and allow comparison. All children were Italian native speaker. See breakdown of participants’ characteristics in Table 1.

**Table 1:** Participants’ characteristics by mean age (in years), age range and gender. Descriptive statistics (mean, standard deviations for accuracy rate and score range) for the Italian vocabulary and nonverbal reasoning test. SDs are in brackets.

	Mean age (*SD*)	Age range	Girls	Italian vocabulary mean score (*SD*) and score range	Nonverbal reasoning mean score (*SD*) and score range
Younger children (n = 44)	8.40(*0*.*53*)	7–8	16	26.18(*2*.*44*) [0–30]	12.75(*5*.*20*) [0–25]
Older children (n = 45)	9.95(*0*.*57*)	9–10	15	27.53(*3*.*03*) [0–30]	13.42(*3*.*24*) [0–25]

Children came from a public school located in Northern Italy, within an urban environment and mixed socio-economic background. All children had normal or corrected-to-normal vision. The study was conducted in accordance with the Ethical Standards laid down in the 1964 Declaration of Helsinki and the standard ethical procedures recommended by the Italian Psychological Association (AIP). The study was reviewed and approved by the IRB (ethical committee) of the University of Pavia/IUSS before the study began. Written informed parental consent (as well as oral informed child assent) was obtained prior to participating, according to the ethical norms in our university.

Children were administered two tasks to assess general cognitive abilities (see following method sections for full description). Descriptive statistics for the two general cognitive abilities administered to the two age groups are displayed in Table 1. Analyses on the accuracy scores (independent sample t-tests) showed age-related differences in the vocabulary test, *t(*87) = 2.09, *p* = .04, with older children better scoring than younger children, but no differences in the visuospatial reasoning test, *t(*88) = 1.02, *p* = .31.

## Materials and procedures

In order to verify that children’s general cognitive performance adhered to the average for their age, they were presented with two measures: a standardized Italian vocabulary test and a nonverbal reasoning test. In particular, the vocabulary can be taken as an index of crystallized intelligence, whereas the nonverbal reasoning test is held to measure fluid intelligence.

In addition, a computerized letter updating task was administered. The vocabulary test and the nonverbal reasoning test were administered in a classroom-based group session. The updating task was administered individually at school, in a quiet room. The group session lasted on average 15 minutes, and the updating task lasted about 20–25 minutes. The two sessions were non-consecutive, in order to avoid possible fatigue effects.

### Italian vocabulary and nonverbal reasoning

The vocabulary and nonverbal reasoning subtests, taken from the Primary Mental Aptitude Battery [32] were presented to the whole class group during a school day; both have a four alternative multiple-choice structure. The vocabulary subtest has 30 items and the nonverbal reasoning subtest, 25 items. Participants had time constraints for both subtests; specifically, 5 minutes for the vocabulary and 6 minutes for the nonverbal reasoning.

### Letter updating task

The task we used in the current paper was described in detail previously, in [14]. As in [14] the stimuli were sub-lexical units between two consonants of the Latin alphabet. The association was based on LTM consonant representation; that is, on the basis of their combined frequency in the spoken Italian language. We evaluated this from the lexicon of frequency of Italian spoken language [33], a corpus of about 490,000 lemmas collected in four main Italian cities, emerging from different subgroups of discourse. High and low frequency lemmas were selected. Low frequency ranged from 0 to 2 (i.e., lemmas with less than 3 occurrences in the corpus). High frequency lemmas had at least 3 occurrences in the corpus.

Next, we inferred strong and weak sub-lexical associations between consonants, based on the lemmas’ frequency. That said, we should note there is no specific frequency information for consonant couples, only for lemmas of the corpus. So, for example, from the lemma “*ardere”* which is low frequency, we inferred the low frequency sub-lexical association “*rd*”. In addition, low frequency associations, typically, were in the middle of the lemma, whereas high frequency lemmas were at the beginning of the lemma. In addition, we checked the corpus to find occurrences of low frequency sub-lexical associations in different lemmas, in order to preclude their presence in high frequency lemmas. We included in the low frequency sub-lexical associations those one occurring in low frequency lemmas only.

We employed the following set of consonants: B C D F G H L N P R S T. Strong associations were: T-R, S-P, P-R, N-T, B-R, C-H, G-R, F-R. Weak ones were: F-L, S-N, G-H, P-S, G-L, R-D, N-D, L-T. Strong and weak associations between consonants were controlled in order to avoid obviously familiar or meaningful couplets. Each association was legal, and thus possible at the sub-lexical level of the Italian language [14].

As described in [14] and in order to avoid ceiling (i.e., with two items) or floor effects (i.e., with four items), we used memory sets composed of three letters (i.e., triplets), which have been established as being within average memory span [34]. Some letters were overrepresented relative to others, but we controlled for this bias by randomizing these across association strengths. Further, the position of the sub-lexical unit within the triplet (i.e., in positions *1/2* or *2/3*) was randomized between trials. We did not control for potential position effects, as it was shown in a previous experiment that position did not interact with either updating or strength (see [14], Experiment 2)

**Fig 1:**
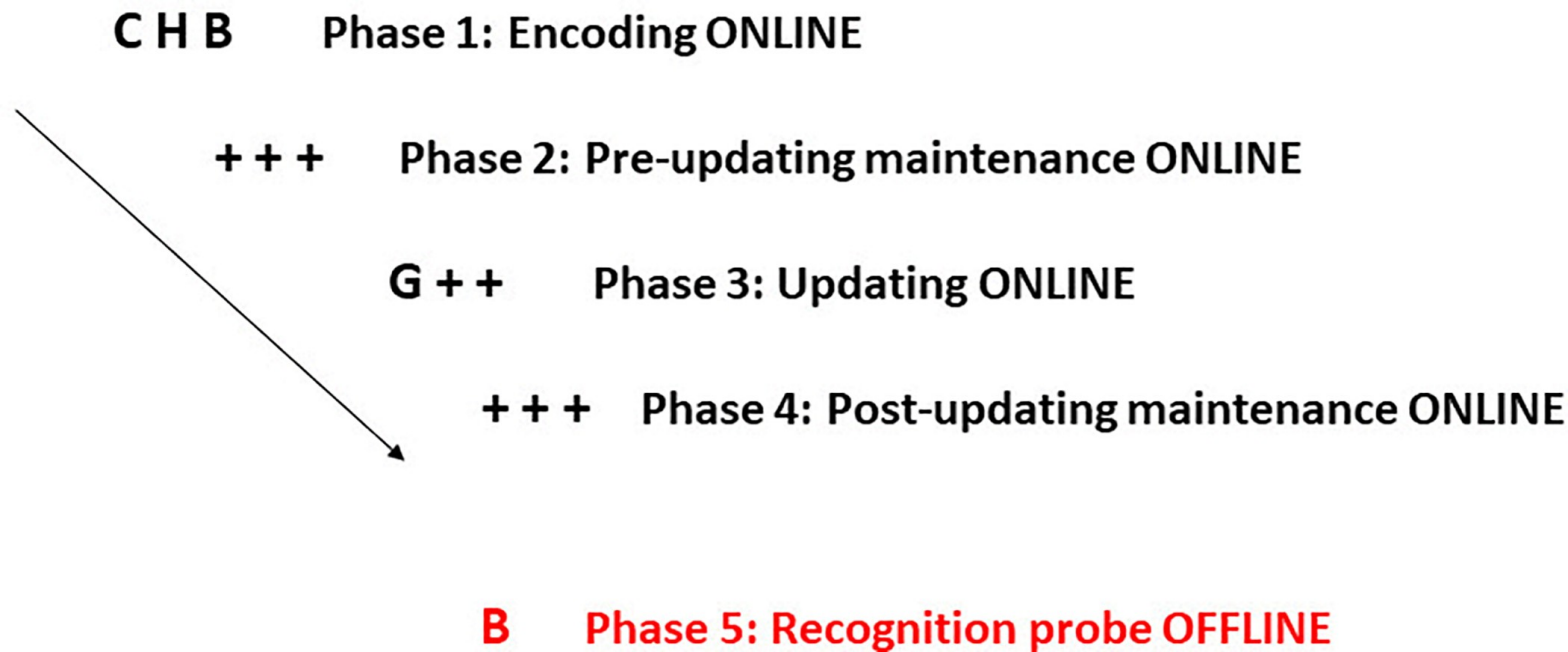
An example of a trial from the letter updating task (strong-to-weak). After encoding the first triplet (*CHB*), participants had to maintain it actively in memory (pre-updating maintenance process: + + +). Next, they were instructed to update part of the association, that is, to remove the item *C* and substitute the *G*. Thus, the triplet they were now maintaining was *GHB*. Lastly, they had to maintain the recently updated triplet (post-updating maintenance process). At recognition, a single red probe was displayed: here, participants had to recognize if the probed item belonged to the most recent studied/updated item or not. In the example, a target probe was presented (*B*), to which they had to give a positive answer.

The third letter of each triplet was another consonant, which was always unrelated to the other two. Specifically, the link between the sub-lexical unit and the third letter was always linguistically impossible in Italian (e.g., see example from Fig 1 where C-H is a strong association, and the link between H and B (H-B) is impossible in Italian). This was done in order to avoid any LTM (strong or weak) or some other meaningful way association between these letters.

### Design and analyses

A three factor mixed design was implemented: Strength and Phase were within-participants factors, and Age group between-participants. The variable Strength had two levels: strong-to-weak and weak-to-weak. ‘Strong-to-weak’ represented associations between letters where the association was strong at encoding, but modified with a weak one upon updating (e.g., from C-H to G-H). Weak-to-weak represented associations between letters occurred where the association was weak at encoding and updated with another weak association (e.g., from P-S to P-R). For each trial, we considered two main phases of encoding (i.e., studying/encoding the initial triplet), and updating (i.e., partial into the triplet). Although the trial was constituted of four phases, only encoding and updating phases (i.e., phases that produce effects on RTs, see [31]) were entered into the analysis.

In addition, to make the task less predictable and ensure participants were engaged, we included several control trials (approximately 20% of the total number). Here, no updating occurred, and maintenance alone was required throughout the trials. These data were not included in further analyses, but were checked to ensure that all updating trials had longer RTs than controls (*p* < .05 for each comparison; control vs. strong-to-weak, and weak-to-weak; [14]).

### Procedure

Procedure was described in detail previously [14, 26]. The task was administered on a standard PC and consisted of four phase subject-paced trials, where participants pressed the spacebar to start each trial, and after each phase, in order to proceed with the task.

In each phase, triplets were always displayed in the centre of the screen. Each trial started with an encoding phase (Phase 1; see an example with letters in Fig 1, where a strong-to-weak association is represented), where participants had to memorize the first triplet of consonants (e.g., C-H-B). A pre-updating maintenance phase followed (Phase 2), where three pluses were displayed; this indicated that the previously encoded triplet had to be actively maintained. Then, at updating (Phase 3), participants had to substitute the no-longer-relevant information (here, C) with newly relevant information (here, G). Concurrently, they needed to maintain previously relevant detail (here, H-B), thus, updating the triplet (i.e., from C-H-B to G-H-B). Finally, a post-updating maintenance (Phase 4) ended the sequence, to control for recency biases. See also Fig 1.

Only one letter of the triplet had to be updated; this letter could be presented in any position of the triplet (i.e., left letter, right letter, or center). Position was balanced across trials, and only new consonants were presented across each phase. When a consonant did not change, a plus symbol was presented, in order to encourage active maintenance of previously encoded/memorized information.

At the end of each trial (Phase 5), participants were presented with a probe recognition task: a single red consonant was displayed in the centre of the screen. Here, they had to indicate whether this belonged to the most-recently studied triplet or not. They responded by pressing one of two keys on the keyboard; one (M for *Yes*) for target probes requiring a positive answer (i.e., belonging to the final triplet of the trial); another one (Z for *No*) for probes requiring a negative answer (i.e., not previously presented in the trial. For these, we included both lures i.e., (probes encoded in the trial, then substituted at updating step) and negative probes (i.e., probes not presented in that trial), mixed within the trial. Half the probes were targets (50%); the other half was equally shared between lures (25%) and negative probes (25%).

Afterwards, each participant was presented with a practice block of eight trials to familiarize themselves with the task. One hundred and twenty trials were then presented shared equally in four blocks. We recorded subject-paced RT at each of the four phases, in addition to probe recognition accuracy at Phase 5.

## Results and discussion

### Updating task: Accuracy and data treatment

Participants performed accurately on an average of 92.80% of trials. As expected, participants were very good in completing the task and very few errors were produced. Accuracy was analysed to verify adequate performance, but the main focus of the analysis was on RT. We ran a mixed 2 x 3 ANOVA, with Strength (weak-to-weak, strong-to-weak) as within-participants factor and Age Group (younger children, older children) as between-participants factor on mean accuracy rates of target, lures and negative responses. A main effect of Age Group reached significance, *F*(1, 87) = 8.38, *p* = .005. Accuracy rate was significantly lower in younger children (116/120 correct trials) than in older children (118/120 correct trials). Only subject-paced RTs for trials that ended with correct probe recognition were analysed. Trials with RTs of less than 150 ms, or exceeding a participant’s mean RT for each condition by more than three intra-individual standard deviations, were considered outliers, and therefore excluded from further analyses (3.92%).

In addition, updating measures (in particular, indexes of RT at the updating step), were highly inter-correlated, suggesting good reliability of the task. In particular, RTs for weak-to-weak associations were strongly correlated, *r* (89) = .84, *p* < .001, to RTs for strong-to-weak.

### Overview of the statistical analyses

We used a mixed-effects model approach to test our hypotheses; the most important advantage of such models is that they allow simultaneous consideration of all factors that may contribute to understanding the structure of the data [35]. Raw RTS were logarithmically transformed to normalize them. These factors comprise not only the standard fixed-effects factors controlled by the experimenter (in our case, age group and strength) but also random-effects factors; that is, factors whose levels are drawn at random from a population (in our case, children). To test the effect of age group (younger children, older children) and strength (strong-to-weak, weak-to-weak) on the variables of online RT, and offline RT three mixed-models were used: one for online RT (with encoding and updating phases as additional factors), another one for RT of correctly detected target probes, and a third for RT of correctly rejected lures. See specific details in the subsections below.

All analyses were performed using the R software [36]; for generalized mixed-effect models, the R package lme4 was used [37]; and the lmer test package was used to obtain Type III ANOVA Tables. Results for each dependent variable are presented below. For planned comparisons, Tukey correction was used to control the Type I error rate.

### Online RT analyses

A linear mixed-effects model was constructed with 3-way interactions between Age Group (younger children, older children), Strength (strong-to-weak, weak-to-weak), and Phase (encode, update). The model revealed a significant effect of Age Group, *F* (1, 87) = 8.11, *p* = .006. Overall, older children (*M* = 2709.26 ms, *SD* = 67 ms) were faster than younger children (*M* = 2960.22 ms, *SD* = 66 ms). Moreover, Strength affected the online processing, *F* (1, 261) = 5.71, *p* = .01; strong-to-weak associations (*M* = 2898.36 ms, *SD* = 65 ms) were hardly updated than weak-to-weak ones (*M* = 2768.30 ms, *SD* = 68 ms).

**Fig 2:**
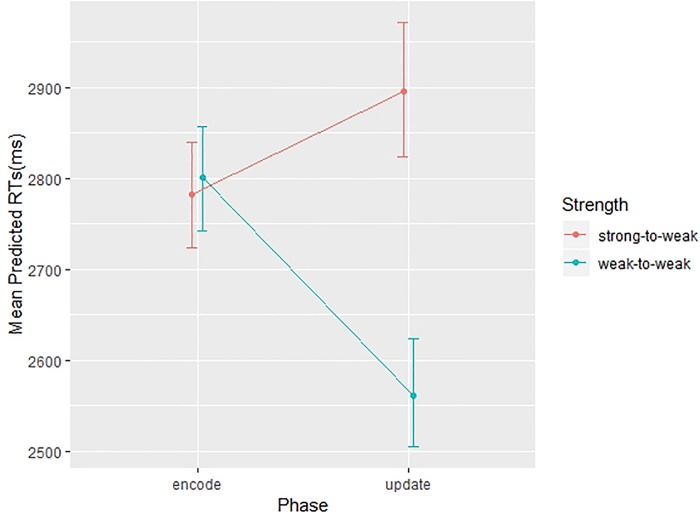
Plot representing the effects of Strength on the two Phases of encode and update. Plot dots represent mean predicted RTs (ms) and bars represent 95% CIs.

In addition, the Phase by Strength interaction reached significance, *F* (1, 261) = 7.18, *p* = .008. Post-hoc comparisons showed no differences at encode across associations, *t(*261) = -0.21, *p* = .83; in contrast, at updating, strong-to-weak associations showed longer RTs compared to weak-to-weak associations, *t(*261) = 3.59, *p* = .004, as shown in Fig 2. No other interaction reached significance.

### Offline RT analyses: Target probes

A linear mixed-effects model was constructed with 2-way interactions between Age Group (younger children, older children) and Strength (strong-to-weak, weak-to-weak). The model revealed a significant effect of Strength, *F* (1, 87.353) = 11.13, *p* = .001. Indeed, we found significantly longer RTs for correct recognition of a target probe from strong-to-weak associations (*M* = 2058.33 ms, *SD* = 58 ms), compared to weak-to-weak associations (*M* = 1867.85 ms, *SD* = 43 ms). No other effect reached significance.

### Offline RT analyses: Lures

First, we conducted a control analysis with Strength (weak-to-weak, strong-to-weak), and Probe (lure, negative) as within-participant factors and Age Group (younger children, older children) as between-participant factor, for lures vs. negative probe RTs. Importantly here, we found a main effect of Probe, *F*(1, 87) = 8.61, *p* = .004, showing longer RTs to recognize and respond to lures (*M* = 2395.68 ms, *SD* = 52 ms) than to negative probes (*M* = 2208.06 ms, *SD* = 44 ms).

In addition, to test our hypotheses more specifically, a linear mixed-effects model was constructed with 2-way interactions between Age Group (younger children, older children) and Strength (strong-to-weak, weak-to-weak) and was run on lures only, as these represent a measure of the ability to inhibit irrelevant information once completed the updating task. The model revealed a significant effect of Age Group, *F*(1, 85.250) = 16.92, *p* < .001. In addition, we found a main effect of Strength, *F*(1, 87.394) = 45.75, *p* < .001.

**Fig 3:**
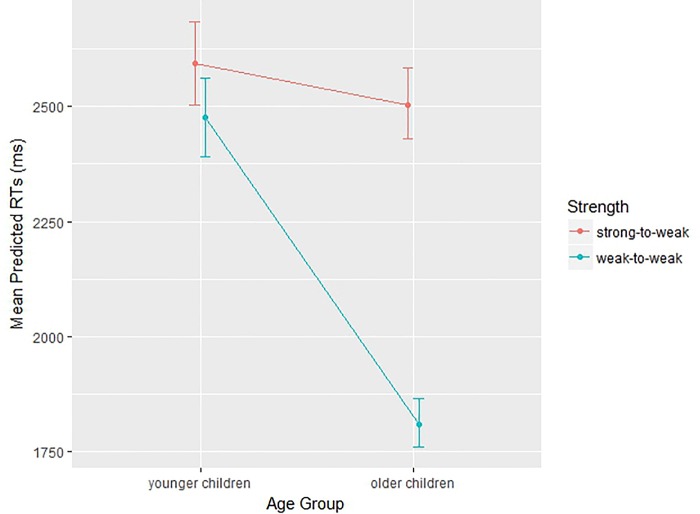
Plot representing the effects of Strength on the two Age groups (younger, older children). Plot dots represent mean predicted RTs (ms) at lure rejection and bars represent 95% CIs.

The two-way Strength by Age Group interaction reached significance, *F*(1, 87.394) = 25.57, *p* < .001. Subsequently, post-hoc comparisons showed that rejection of a lure from a strong-to-weak association needed longer RTs (compared to weak-to-weak association), but only for older children, *t*(87.39) = 8.41, *p* < .001. Rejection of a lure from a strong-to-weak association did not differ from a weak-to-weak condition in younger children, *t*(87.39) = 1.20, *p* = .23, as shown in Fig 3.

We believe our task is mainly based on phonological/orthographic knowledge and less on lexico-semantic knowledge (see also [30]). In fact, in order to engage with the task rapidly and effectively, the child should have developed an automatic access to orthographic/letter form representation. Therefore, we do not predict any specific vocabulary-related effect. However, in order to control for the role of vocabulary in the process examined, we ran the same mixed-effect models, covarying for vocabulary. Overall, the results did not change, showing the same effects and significance levels for both target probes (main effect of Strength, *p* = .002) and lures (Age group, Strength, and two-ways interaction, all *ps* < .001).

## Conclusions

In this study, our aim was to investigate how LTM associations affect updating development. Updating is a complex activity that involves inhibition at different levels such as from the same lists set, or from previous lists [9], with the distinguishing component of the item-removal process [16, 18]. More specifically here, we analysed how the strength of LTM association between items affects updating from a developmental perspective.

Typically, the literature on adults shows enhanced recall for strongly associated items; the stronger the pre-existing association in LTM, the better the performance in WM. For updating, a somewhat different process is indicated (i.e. not only maintenance of information in the short term, but also removal of irrelevant information). In this case, the opposite was shown: the stronger the pre-existing association, the harder it is to dismantle it [26].

In addition, the first notable difference between updating and recall (i.e., slowing of RTs in the former) could be related to the number of cognitive operations required in the task. Indeed, recall involves maintenance of information only; whereas updating entails a further item-removal component. Therefore, it is reasonable to assume that an additional operation (i.e., item-removal) will add a cost in terms of longer processing latencies. However, results comparing updating performance compared to recall have demonstrated the reverse effect; that is a cost rather than a benefit. This difference is likely to be due to the nature of updating, an essential process in adaptation of WM content to new elements. In other words, updating involves integration of new elements, as well as new bindings between elements (after disrupting previous ones), thus inhibiting and removing/substituting irrelevant information [11, 16].

A recent model of updating [9] showed that updating develops via two main components of inhibition, one more related to control of inhibition from same lists; another one of inhibition from previous lists. The former, shows fewer developmental differences, the latter (also called PI control in [9]) shows greater age-related differences. In our view, the task used here with children is suitable for consideration of both components in terms of processing speed (an index useful in studying development via more subtle and fine-grained measurement). In fact, in the current task, each participant needs to maintain information and inhibit it, when no longer relevant, by substituting with new information during the tasks (same list inhibition component). Further, to ensure effective updating, s/he has to control for interference from previously studied items which are no longer relevant (i.e., inhibition from previously studied items set).

In particular, in accordance with [9] model, we found different outcomes consistent with the measures considered. Accordingly, the online RT showed a global age-related effect (older children faster than younger children), but not specific for strength with which letter were associated (in fact, no interaction). This finding could be accounted for, if we consider the development of self-monitoring (i.e., the ability to control one own’s behaviour) in children. That is, monitoring skills develop between 7 to 10 years, and subtle but important improvements are found over the primary school years [38]. Our self-paced task, where the child had to press the spacebar when s/he thinks to have memorized/updated a given mental set, requires a self-judgment of performance from the child him/herself. In particular, it has been shown that children (from 8 years of age) are more accurate in judgment of learning when given after a delay of about 2 minutes, than immediately after study [39]. Thus our task (which requires self-monitoring of learning during the study/updating phases, and immediately after, in order to press the spacebar) might not enhance an appropriate child self-regulation. For this reason, we believe we did not find age-related effects relative to strength for self-paced RTs and thus, failed to replicate the effects found with adults [14, 26].

Conversely, for offline inhibitory control (i.e., lure recognition), we found more pronounced developmental effects, with significant differences; older children took more time to reject strong lures than weak, whereas no difference was observed for younger children (Fig 3). Therefore, we found that online inhibition component was less affected by developmental change: younger children are able to perform updating tasks successfully. The real challenge in updating (i.e., due to control for previously relevant information) elicits significantly better performance from 10 years onwards. Here, in fact there is no need for self-regulation (i.e., as in the probe recognition task) as the task is not self-paced. The modulation of association strength development in older children (but not in younger) could be well accounted by the development of both lexico-orthographic knowledge and executive mechanisms that can work simultaneously [5, 6].

This finding supports claims that the ability to inhibit irrelevant information is a fundamental mechanism that underlines many other developmental changes [40, 41, 42]. In particular, decreased susceptibility to interference is observable as age increases; 7/8 years olds children were shown to be more susceptible to interference than 9/10 years old [40], as we found in our study. However, we believe the novelty of the current study lies in the specificity of the experimental manipulation. Notably, these results indicated that, from 10 years onward, children found highly familiar stimuli (such as letters) more intrusive and difficult to control when strongly associated. Therefore, although we find that older children are less susceptible to interference, it seems that they are more sensitive to strong and weakly associated stimuli, similarly to performance in adults [14, 26].

Future studies should further investigate any additional benefits/costs in updating strong and weak LTM associations, by also manipulating the strength of the item-association at updating [14]. Through this further manipulation, a more fine-grained examination of the dismantling and recreation of associations during updating would be enabled, including analysis of the relative ease/difficulty of the process. In addition, it could be useful to administer the task to children with specific learning disorders in order to show possible modulation of WM performance by LTM knowledge. Specifically, the task could then be useful to implement ad hoc measures to train children to remediate identified weaknesses, both in educational and clinical settings. We might also speculate that, as we found that strong LTM associations are more difficult to modify, this could in turn indicate the importance of correct support for the child, so that s/he will not act to strengthen incorrect sub-lexical/phonotactic associations. Indeed, it is likely that the more those incorrect associations are reinforced, the harder will be to modify/update them.

In conclusion, the present study demonstrated how WM updating is affected by LTM strength of association in a developmental sample. A significant cost of dismantling and updating strong associations was shown, and this effect was independent from age; all children from 7 to 10 years were comparably sensitive to association strength. In addition, results allowed us to differentiate age-related effects for interference control in updating of strong LTM associations; older children (but not younger) were more susceptible to interference from strongly-associated information.

## Supporting information

S1 Data Dataset online RTs. (XLS)

S2 Data Dataset recognition probe RTs. (XLS)

## References

[1] Cain K., Oakhill J., & Bryant P. (2004). Children's reading comprehension ability: Concurrent prediction by working memory, verbal ability, and component skills. *Journal of Educational Psychology*, 96, 31–42. http://psycnet.apa.org/doi/10.1037/0022-0663.96.1.31.

[2] Cowan N. (2017). The many faces of working memory and short-term storage. *Psychonomic Bulletin & Review*, 24, 1158–1170. 10.3758/s13423-016-1191-6. 27896630

[3] Daneman M., & Carpenter P. A. (1980). Individual differences in working memory and reading. *Journal of Verbal Learning and Verbal Behavior*, 19, 450–466. 10.1016/S0022-5371(80)90312-6.

[4] Palladino P., Cornoldi C., De Beni R. & Pazzaglia F. (2001). Working memory and updating processes in reading comprehension. *Memory & Cognition*, 29, 344–354. 10.3758/BF03194929 11352218

[5] Bathelt J., Gathercole S. E., Johnson A., & Astle D. E. (2018). Differences in brain morphology and working memory capacity across childhood. *Developmental Science*, 21, e12579 10.1111/desc.12579 28748537 PMC5947821

[6] Gathercole S. E., Pickering S. J., Ambridge B., & Wearing H. (2004). The structure of working memory from 4 to 15 years of age. *Developmental Psychology*, 40, 177–190. 10.1037/0012-1649.40.2.177 14979759

[7] Gathercole S. E., & Adams A. M. (1994). Children′ s phonological working memory: Contributions of long-term knowledge and rehearsal. *Journal of Memory and Language*, 33, 672–688. 10.1006/jmla.1994.1032.

[8] Cowan N., Saults J. S., & Blume C. L. (2014). Central and peripheral components of working memory storage. *Journal of Experimental Psychology*: *General*, 143, 1806–1836. http://psycnet.apa.org/doi/10.1037/a0036814. 24867488 10.1037/a0036814 PMC4172497

[9] Carriedo N., Corral A., Montoro P. R., Herrero L., & Rucián M. (2016). Development of the Updating Executive Function: From 7-Year-Olds to Young Adults. *Developmental Psychology*, 52, 666–678. 10.1037/dev0000091 26882119

[10] Morris N., & Jones D. M. (1990). Memory updating in working memory: The role of central executive. *British Journal of Psychology*, 81, 111–121. 10.1111/j.2044-8295.1990.tb02349.x

[11] Schmiedek F., Hildebrandt A., Lövdén M., Wilhelm O., & Lindenberger U. (2009). Complex span versus updating tasks of working memory: The gap is not that deep. *Journal of Experimental Psychology*: *Learning*, *Memory*, *and Cognition*, 35, 1089–1096. 10.1037/a0015730 19586272

[12] Artuso C. & Palladino P. (2014). Binding and content updating in working memory tasks. *British Journal of Psychology*, 105, 226–242. 10.1111/bjop.12024 24754810

[13] Boujut A. & Clarys D. (2016). The effect of ageing on recollection: the role of the binding updating process. *Memory*, 24, 1231–1242. 10.1080/09658211.2015.1091893 27560656

[14] Artuso C. & Palladino P. (2018). How sub-lexical association strength modulates updating: Cognitive and strategic effects. *Memory & Cognition*, 46, 285–297. 10.3758/s13421-017-0764-6. 29047045

[15] Taylor H. A., Thomas A. K., Artuso C. & Eastman C. (2014). Effects of global and local processing on visuospatial working memory. *Spatial Cognition IX*, *Lecture Notes in Computer Science*, 8684, 14–29. 10.1007/978-3-319-11215-2_2

[16] Ecker U. C., Lewandowsky S., Oberauer K. & Chee A. E. (2010). The components of working memory updating: an experimental decomposition and individual differences. *Journal of Experimental Psychology*: *Learning*, *Memory and Cognition*, 36, 170–189. 10.1037/a0017891 20053053

[17] Oberauer K. (2005). Binding and inhibition in working memory: individual and age differences in short-term recognition. *Journal of Experimental Psychology*: *General*, 134, 368–387. 10.1037/0096-3445.134.3.368 16131269

[18] Lewis‐Peacock J. A., Kessler Y., & Oberauer K. (2018). The removal of information from working memory. *Annals of the New York Academy of Sciences*, 1, 1–12. 10.1111/nyas.13714 29741212

[19] Linares R., Bajo M. T., & Pelegrina S. (2016). Age-related differences in working memory updating components. *Journal of Experimental Child Psychology*, 147, 39–52. 10.1016/j.jecp.2016.02.009 26985577

[20] Hulme C., Stuart G., Brown G. D. A. & Morin C. (2003). High- and low-frequency words are recalled equally well in alternating lists: Evidence for associative effects in serial recall. *Journal of Memory and Language*, 49, 500–518. 10.1016/S0749-596X(03)00096-2

[21] Saint-Aubin J., Ouellette D., & Poirier M. (2005). Semantic similarity and immediate serial recall: Is there an effect on all trials. *Psychonomic Bulletin & Review*, 12, 171–177. 10.3758/BF03196364. 15945210

[22] Belacchi C., Benelli B., & Pantaleone S. (2011). The influence of categorical organization on verbal working memory. *British Journal of Developmental Psychology*, 29, 942–960. 10.1111/j.2044-835X.2011.02030.x 21995746

[23] Majerus S. & Van der Linden M. (2003). Long-term memory effects on verbal short-term memory: A replication study. *British Journal of Developmental Psychology*, 21, 303–310. 10.1348/026151003765264101

[24] Monnier C., & Bonthoux F. (2011). The semantic‐similarity effect in children: Influence of long‐term knowledge on verbal short‐term memory. *British Journal of Developmental Psychology*, 29, 929–941. 10.1111/j.2044-835X.2010.02024.x 21995745

[25] Gathercole S. E., Frankish C. R., Pickering S. J. & Peaker S. (1999). Phonotactic influences on short-term memory. *Journal of Experimental Psychology*: *Learning*, *Memory and Cognition*, 25, 84–95. 10.1037/0278-7393.25.1.84 9949710

[26] Artuso C. & Palladino P. (2016a). Modulation of working memory updating: Does long-term memory lexical association matter? *Cognitive Processing*, 17, 49–57. 10.1007/s10339-015-0735-4 26323831

[27] Lendínez C., Pelegrina S. & Lechuga M. T. (2011). The distance effect in numerical-memory updating task. *Memory & Cognition*, 39, 675–685. 10.3758/s13421-010-0047-y 21264591

[28] Lendínez C., Pelegrina S. & Lechuga M. T. (2014). The role of similarity in updating numerical information in working memory: decomposing the numerical distance effect. *Quarterly Journal of Experimental Psychology*, 67, 16–32. 10.1080/17470218.2013.793375 23679060

[29] Nairne J. S. (1990). A feature model of immediate memory. *Memory & Cognition*, 18, 251–269. 10.3758/BF03213879. 2192233

[30] Artuso C. & Palladino P. (2016b). Letter updating is related to reading fluency but not comprehension. *Learning & Individual Differences*, 52, 53–59. 10.1016/j.lindif.2016.10.008.

[31] Artuso C., & Palladino P. (2011). Content-context binding in verbal working memory updating: on-line and off-line effects. *Acta Psychologica*, 136, 363–369. 10.1016/j.actpsy.2011.01.001 21276582

[32] Thurstone L. L. & Thurstone T. G. (1963). Italian edition 1981 *PMA*: *Batteria delle attitudini mentali primarie*, *7–11 anni [PMA*: *Primary mental abilities*, *7–11 years*]. Edizioni Giunti OS: Firenze.

[33] De Mauro T., Mancini F., Vedovelli M. & Voghera M. (1993). Lessico di frequenza dell'italiano parlato [Frequency Lexicon of Spoken Italian Language] Milano: Etas Libri.

[34] Cowan N. (2001). The magical number 4 in short-term memory: a reconsideration of mental storage capacity. *Behavioral Brain Sciences*, 24, 87–114. 10.1177/0963721409359277 11515286

[35] Baayen R. H., Davidson D. J., & Bates D. M. (2008). Mixed-effects modeling with crossed random effects for subjects and items. *Journal of Memory and Language*, 59, 390–412. 10.1016/j.jml.2007.12.005.

[36] R Development Core Team. (2010). R: A language and environment for statistical computing. Vienna, Austria: R foundation for statistical computing.

[37] Bates D. M., & Maechler M. (2010). *lme4*: *Linear mixed-effects models using S4 classes*. R package version 0.999375-36/r1083.

[38] Schneider W. (2008). The development of metacognitive knowledge in children and adolescents: Major trends and implications for education. *Mind*, *Brain*, *and Education*, 2, 114–121. 10.1111/j.1751-228X.2008.00041.x

[39] Schneider W., Visé M., Lockl K., & Nelson T. O. (2000). Developmental trends in children's memory monitoring: Evidence from a judgment-of-learning task. *Cognitive Development*, 15, 115–134. 10.1016/S0885-2014(00)00024-1.

[40] Dempster F. N. (1992). The rise and fall of the inhibitory mechanism: Toward a unified theory of cognitive development and aging. *Developmental Review*, 12(1), 45–75. 10.1016/0273-2297(92)90003-K.

[41] Hale S., Bronik M. D., & Fry A. F. (1997). Verbal and spatial working memory in school-age children: developmental differences in susceptibility to interference. *Developmental Psychology*, 33, 364–371. http://psycnet.apa.org/doi/10.1037/0012-1649.33.2.364. 9147843 10.1037//0012-1649.33.2.364

[42] Nigg J. T. (2000). On inhibition/disinhibition in developmental psychopathology: views from cognitive and personality psychology and a working inhibition taxonomy. *Psychological Bulletin*, 126, 220–246. http://psycnet.apa.org/doi/10.1037/0033-2909.126.2.220. 10748641 10.1037/0033-2909.126.2.220

